# Retirement and perceived social inferiority strongly link with health inequalities in older age: decomposition of a concentration index of poor health based on Polish cross-sectional data

**DOI:** 10.1186/s12939-016-0310-3

**Published:** 2016-02-04

**Authors:** Zuzanna Drożdżak, Konrad Turek

**Affiliations:** Swiss Tropical and Public Health Institute, Socinstrasse 57, 4051 Basel, Switzerland; University of Basel, Basel, Switzerland; Centre for Evaluation and Analysis of Public Policies, Jagiellonian University, ul. Grodzka 52, 31-044 Krakow, Poland

**Keywords:** ESS, Inequality, SES, Social determinants of health, Subjective social position, Aging

## Abstract

**Background:**

Identifying mechanisms that generate and sustain health inequalities is a prerequisite for developing effective policy response, but little is known about factors contributing to health inequalities in older populations in post-transitional European countries such as Poland. Demographic aging of all populations requires new and deeper insights.

**Methods:**

Data came from the Polish edition of the cross-sectional European Social Survey, Wave 6 (2012). Logistic regression was applied to identify socioeconomic factors relevant to self-assessed health in a population aged 45 or over. Decomposition of a concentration index provided information about the distribution of health-relevant demographics and social characteristics along a socioeconomic continuum, and their contributions to observed health inequalities.

**Results:**

Overall, 17.4 % of respondents aged 45 or over assessed their health as poor or very poor. Predictors of poor health included income insufficiency, disability or retirement, retirement, low social activity, and social position. A steep socioeconomic gradient in self-assessed health in Polish population was found. The primary contributor to the observed health inequality (as summarized by concentration index) was income, followed by labor market situation, particularly retirement. Self-assessed place in society contributed to overall inequality, scoring similarly to social activity. Contributions from age and education were moderate but non-significant, gender was negligible, and chronological aging explained neither poor health nor socioeconomic health inequalities.

**Conclusions:**

Although elderly people represent a particularly vulnerable group, their disadvantages are associated with social rather than natural causes. Policies addressing health inequalities in aging populations must provide systemic opportunities for maintaining good health. Transitioning to retirement is a critical entry point for policy action that stimulates social engagement and maintains self-esteem of older people.

## Background

Creating effective policy responses to growing health disparities is difficult since systemic mechanisms that trigger and sustain socioeconomic inequalities in health differ across populations and societies. An important context of research on health inequalities is nowadays population aging. The growing share of older people in most developed countries influences the volume and structure of demand for healthcare, as well as generates new social challenges. To create a successful health policy for aging populations, we must understand not only the determinants of health and disease, but also sources of health inequalities during older age.

When discussing deterioration of health status and functional capacities, chronological age is a fundamental dimension of analysis. Health decline during older age is considered a normal consequence of aging, yet the relationship between age and biological aspects of human body condition is not as straightforward as it seems. Most researchers find a decreasing trend in physical abilities, particularly in strength, agility, sensory abilities, and speed as a consequence of aging [1–3], but aging does not have to result in decreases in functional capacities or health-related quality of life, especially in the young-old (i.e. aged about 55–75 years). For example, based on 8-year panel data from Canada, Asakawa [4] estimates that the overall decline in health with age is, on average, negligible until the age of 60, at which time it accelerates. Other studies suggest a steady decrease in self-assessed health in populations at middle age [5]. From a meta-analysis of the relationship between age and workers’ health, Ng found a modest decline in clinical indicators of physical health in older workers (e.g., blood pressure, cholesterol, and body mass index) [6]. However, the effect of workers’ ages was non- significant for self-reported physical and mental health.

The most important conclusion from many studies of health decline with age is that the speed of the process differs between individuals, which results in increasing socioeconomic health inequalities in older age groups [8–10]. People with higher socioeconomic status (SES) live, on average, longer and in better health. Low SES acts as a clustering factor for multiple health disadvantages [10], including unhealthy, hazardous lifestyles, low access to healthcare, psychological strain, adverse working and living conditions, and others [11, 12]. High SES in turn provides better opportunities to mitigate risks and avoid exposures.

The consequences of health inequalities in older age groups gained particular importance in the context of population aging, from which emerge two important questions. The first is whether population aging will stimulate growth in health inequalities. It appears there is no simple relationship between these two phenomena. Increasing life expectancy extends the time for inequalities to rise due to the accumulation of advantages and disadvantages [13–17], but among the oldest old people (i.e. about 75+), inequalities decrease in comparison to those who are younger. Age acts as a leveler through two mechanisms: a) the general decline in physical abilities among the oldest cohorts, even those with high SES [16], and b) selective mortality, which is higher among those with the lowest health [10].

The second question stems from the fact that most developed countries are considering or have already initiated actions aimed at increasing the retirement age [17]. What are their consequences to health inequalities? Some authors debate whether people are sufficiently healthy to work longer (cf. [18]). Majer et al. [8] suggest that rising retirement ages disproportionally affects people at lower and higher socioeconomic levels because of their disparate health statuses and subsequent work abilities. A related issue is health consequences of the transition from worker to pensioner, and the literature remains inconclusive about this (cf. [19]). Many studies emphasize negative consequences of retirement on physical, mental, and self-assessed health [7], suggesting reductions in psychological wellbeing [20, 21], increases in difficulties associated with daily activities, increases in illnesses, declines in mental health [22], and decreases in cognitive functionality [23]. These adversity of these effects deepen with the amount of time spent in retirement [24], yet retirement is not necessarily a negative experience, especially since it increases leisure time and provides opportunities to pursue personal interests [20, 25–29]. There is also a possibility that the adverse health events observed during the retirement had been experienced already before [24]. In some cases, and most likely more often amongst workers coming from disadvantaged social classes, [7] health problems might have in fact motivated labor market disengagement in the first place. Claiming that retirement causes health declines rather than health declines causing retirement might be an example of incorrect reverse causal conclusions. Most likely, the effect of retirement on self-perceived health is moderated strongly by individual and contextual characteristics such as perception of control over one’s life, education, receipt of a disability pension, family structure, work environment, satisfaction at work, labor-market instability, and timing of retirement [28, 30–32]. These in turn relate strongly to socioeconomics.

This study identifies factors relevant to self-assessed health and that contribute to health inequalities in the aging Polish population. Decomposition of socioeconomic inequalities into underlying factors was so far performed using data from very different populations around the globe [32–39]. We used this well-established method to perform a similar decomposition on Polish data. In Poland, elderly people bear the largest burden of health inequalities due to the dual nature of their disadvantage—being both more susceptible to disease and socioeconomically disadvantaged [40]. Such vulnerability is to a large extent a consequence of political and economic transformations from communism to a market economy during the early 1990s which introduced new rules of social organization of life in countries located in Central and Eastern Europe bringing differential consequences for various generations. Young people benefited from the new life-course regime, which enhanced educational opportunities [41, 42]. Yet income inequalities rose sharply as the labor market adopted capitalist logic, which resulted in significant unemployment [43], and pushing older workers out [42]. All of these circumstances—changes to political systems and institutional contexts, rising income inequalities and market competition, and forced disengagement—are likely relevant to the health of elderly people, encouraging health inequalities among them. Therefore, Poland is a vivid example of structural and societal health disparities in an elderly population in comparison to more stable, Western economies.

## Methods

### Source of data

Data for this study were collected in 2012 as a part of the Polish Edition of European Social Survey (ESS), Round 6. The random sample is representative of a non-institutionalized population aged at least 15 years old and residing in Poland. The study used two-domain probability sampling. Residents of big cities (i.e., over 500,000 inhabitants) were selected using simple random selection, while the remaining participants were pulled from a 2-step, clustered sample from smaller towns and villages. The response rate for the primary questionnaire was 75 %. More details of the study’s design can be found elsewhere [44]. In order to better understand how health inequalities might develop over the life course we included a broad segment of population (people aged 45 or over) into our analyses. Since retirement is important focus of this study, we wanted that the sample consisted of those in pre-retirement age, those eligible to retire and those who retired. At the time of data collection the eligible retirement age in Poland was 60 for women and 65 for men, however broad opportunities for early retirement result in average effective retirement age being 5 and 3 years shorter, respectively [45] and many relatively young retirees. We used all available cases for univariate analyses (1035 cases) and complete cases for multivariate analyses (741 cases). Appropriate weights were used during all analyses to account for sampling error, non-response bias, and selection probability.

### Self-assessed health

Poor self-assessed health (SAH) was the dependent variable. Respondents evaluated their health as very good, good, fair, poor, or very poor while answering the question “How is your health in general?” We use a dichotomized version of this variable, where *poor* or *very poor* SAH indicated a poor health status. Contrary to clinical biomarkers, SAH is contingent on individual predispositions and cultural patterns [46, 47]. It links closely with quality of life, psychological wellbeing, depression, and anxiety [27, 28]. Nevertheless, SAH correlates strongly with objective health assessments and health status indices, including measures of physical and functional health [48]. High validity, combined with ease of data collection, makes SAH one of the most common indicators of overall health status.

### Socioeconomic status

An index of socioeconomic status was constructed using categorical, principal-component analysis [49] using the full sample of respondents, regardless of age. We used a classic sociological approach, combining information concerning decile of income (10 points), education (measured on 27-point ISCED scale), and occupation (measured using ISCO08 international classification of occupations). This statistical method allowed non-linear optimal transformation of categorical data and reduction of information contained in a set of variables to a few principal components. The first principal component preserved 74 % of the original variance, and tests of reliability and stability of the solution yielded favorable results. This first principal component was used during subsequent analyses as a measure of socioeconomic status and a stratification variable.

### Covariates

Explanatory variables for health and health inequalities included age, income sufficiency, level of education, labor-market participation, level of social activity, and self-assessed position in society. We introduced age to the model as a set of dummy variables with the youngest category as a reference. The sample consisted of various age cohorts, but such a design was shown to estimate life-course aging well [5]. Income data were obtained by asking respondents how they felt about their current household income. Having analyzed distributions, we dichotomized this variable into those who find it either difficult or very difficult to live on their present incomes and those who either cope or live comfortably on their present incomes (the latter being a reference category). Education was recoded from an ISCED scale into 3 categories: lower, middle, and higher levels of education. We distinguished 3 categories of economic activity: being active in the labor market (i.e., employed or unemployed), being permanently sick or disabled, and being retired. Self-assessed position in society was measured, using an 11-point scale, by asking respondents to estimate their place in society, given that zero represented the bottom and 10 the top of society. Outliers choosing the lowest or highest positions were removed since other socioeconomic characteristics contradicted such extreme judgements. Subjective socioeconomic position correlates strongly with both physiological and psychological measures of health [50, 51], and we used it as an indicator of perceived social superiority/inferiority.

### Statistical analysis

To assess which factors relate to health, we used logistic regression, in which SAH was the dependent variable and covariates were independent variables. To assess factors that contribute to health inequalities, we used the concentration index (CI) with Erreygers’ correction, a quasi-absolute measure appropriate for binary health outcomes [52]. CI is a numerical representation of a concentration curve, obtained by plotting the distribution of poor health on the x-axis against a distribution of a socioeconomic variable on the y-axis. A negative CI indicates concentration of disease in lower levels of society, and a positive CI indicates concentration of disease among those who are more affluent [53].

It is possible to decompose the CI into contributions generated by pre-specified explanatory factors and unexplained residuals [32]. The decomposition of inequality was conducted using a regression model (logit model), yielding estimation of a) covariate-specific concentration, b) elasticity, the direction and magnitude of the relationship between a variable and health, and c) a percentage contribution of a covariate to overall inequality, summarized by the CI [32]. Covariate-specific concentration indices show distribution of a factor along the socioeconomic continuum. Elasticity informs about the probability of poor health. In the case of ratio-scale variables, a classic economic interpretation is possible (e.g., what percent change in chances of a disease is predicted by a 1 % increase in a covariate). In the case of dummy variables, the sign of the elasticity informs about whether there is a health benefit or disadvantage associated with belonging to a category, in comparison to a reference category. A product of covariate-specific CI and its elasticity informs about what concentration of a disease is produced by a covariate alone, presented in absolute terms. Division of the absolute contribution by the overall magnitude of health inequality, as measured by Erreygers CI, produces a percentage contribution of a covariate to overall health inequality. The strength of the method lies in being able to analyze the contribution of multiple correlates to the joint distribution of poor health and socioeconomics. It provides unique insights into sources of health inequality, rather than sources of poor health and socioeconomic inequality separately.

## Results

### Factors associated with health

Overall, 17.4 % of respondents aged 45 or over assessed their health as poor or very poor. A list of demographics that associate with health appears in Table 1.

**Table 1:** Crude and adjusted association between poor self-assessed health and its predictors

Covariates	Number	% with poor or very poor health	OR unadjusted		OR adjusted***	
			*p*-value	OR (95 % CI)	*P*-value	OR (95 % CI)
Gender						
*Man*	451	15 %	-	1	-	1
*Woman*	582	19 %	0.124	1.30 (0.93–1.80)	0.618	1.11 (0.74–1.65)
Age						
*45–59*	126	6 %	-	1	-	1
*50–54*	165	11 %	0.098	2.14 (0.87–5.26)	0.418	1.49 (0.57–3.92)
*55–59*	188	8 %	0.349	1.55 (0.62–3.88)	0.388	0.63 (0.22–1.80)
*60–64*	177	14 %	0.029*	2.65 (1.11–6.35)	0.696	0.81 (0.28–2.33)
*65–69*	116	30 %	0.001**	7.23 (3.07–17.05)	0.363	1.63 (0.57–4.70)
*70–74*	99	24 %	0.001**	5.46 (2.25–13.26)	0.600	1.34 (0.45–3.98)
*75–79*	86	33 %	0.001**	8.20 (3.39–19.86)	0.167	2.16 (0.76–6.43)
*80+*	77	36 %	0.001**	9.47 (3.88–23.11)	0.460	1.53 (0.50–4.72)
Education						
*Higher*	119	4 %	-	1	-	1
*Lower education*	696	21 %	0.001**	6.23 (2.48–15.63)	0.233	1.84 (0.68–4.99)
*Middle*	219	13 %	0.017*	3.32 (1.24–8.91)	0.508	1.44 (0.49–4.17)
Income						
*Not difficult on present income*	607	10 %	-	1	-	1
*Difficult on present income*	426	29 %	0.001**	3.84 (2.73–5.42)	0.001**	2.77 (1.85–4.14)
Labor market status						
*On labor market*	486	6 %	-	1	-	1
*Permanently disabled*	9	61 %	0.001**	24.41 (5.89–101.14)	0.011*	10.63 (1.73–65.31)
*Retired*	538	27 %	0.001**	5.66 (3.73–8.58)	0.001**	4.24 (2.25–7.99)
Level of social activity						
*Average or higher than others in the same age*	792	13 %	-	1	-	1
*Lower than others in the same age*	202	33 %	0.001**	3.18 (2.22–4.54)	0.001**	2.42 (1.58–3.70)
Self-assessed social position (ordinal, 9 points, low to high)	989	-	0.001**	0.733 (0.66–0.82)	0.007*	0.85 (0.748–0.96)

*p = < 0.05, **p = < 0.001

***Adjusted for: gender, age, level of education, income, labor market status, level of social activity and self-assessed social position

Shares of those reporting poor health in multiple age categories, and unadjusted ORs (Odds Ratios), suggest generally that age is associated with health, albeit not monotonously. For example, people aged 65 to 69 reported poor or very poor health 7 times more often than those aged 45 to 49 (OR 7.232, *p* < 0.001), and people in all age categories were, on average, less healthy than the youngest group. There was no difference between men and women in terms of health, but many other social characteristics did associate strongly with health. Those with lower levels of education were 6 times more likely (OR 6.225, *p* < 0.001), and those with middle levels of education 3 times more likely (OR 3.319, *p* < 0.05), to report poor health. Income insufficiency and low social activity predicted poor health (OR 3.384, *p* < 0.001 and OR = 3.176, *p* < 0.001, respectively). People who graded themselves low in terms of social position had low health, and as self-assessment of social position rose by one point, the chances of reporting poor health diminished by nearly 30 % (OR 0.733 for a 9-point scale, *p* < 0.001). Finally, labor-market situation was relevant to health. Poor health in permanently disabled people who are unable to work was confirmed (OR 24.41, *p* < 0.001) but being retired also increased the chances of reporting poor health more than 5 times (OR 5.658, *p* < 0.001).

The list of adjusted ORs unveils a quite different pattern. When other covariates were controlled for, the association between education and health was alleviated, as was the association between age and health. However, some covariates maintained associations with health. For example, higher assessments of socioeconomic status continued to provide a health benefit, albeit smaller than the crude OR suggests (OR 0.845, *p* < 0.001). The strength of association (i.e., OR) between financial difficulties and health diminished but remained significant (crude OR 3.842 dropped to adjusted OR 2.767, *p* < 0.001), and so for low social activity (crude OR 3.176 dropped to adjusted OR 2.418, *p* < 0.001). When all other factors were controlled for, being a pensioner was associated with a 4-fold increase in the chances of poor health.

### Factors associated with socioeconomic gradient in health

Health was distributed unequally along the socioeconomic continuum, to the disadvantage of people with lower socioeconomic statuses. In the lowest quintile of socioeconomic status, 31 % considered their health poor or very poor, and in the highest quintile of socioeconomic status, this share decreased to 8 % (results not shown). The CI of poor self-assessed health reached −0.169 (*p* < 0.001), suggesting a health gradient to the disadvantage of lower socioeconomic groups.

Although the previously shown analyses elucidate factors relevant to health, they are insufficient to identify factors which contribute to socioeconomic inequalities of health. A general rule suggests health inequality occurs when a group accumulates both socioeconomic advantage and good health simultaneously (or analogously, socioeconomic disadvantage and poor health). This reasoning was implemented during decomposition of the CI of poor self-assessed health (Table 2).

**Table 2:** Decomposition of socioeconomic inequalities in health in the older population in Poland, 2012

Covariates	Model 1				Model 2			
	residual = −0.0011; pseudo R^2^ = 0.1758				residual = −0.0005; pseudo R^2^ = 0.2069			
	*p*-value	Elasticity	Concentration index	% contribution	*p*-value	Elasticity	Concentration index	% contribution
Gender	-	-	-	−0.13 %	-	-	-	0.02 %
* (R = Man)*	-	-	-	-	-	-	-	-
* Woman*	0.689	−0.014	−0.022	[−0.13 %]	0.997	0.002	−0.025	-
Age	-	-	-	21.25 %	-	-	-	9.45 %
* (R = 45-49)*	-	-	-	-	-	-	-	-
* 50–54*	0.299	0.027	0.119	[−1.30 %]	0.650	0.014	0.118	[−0.68 %]
* 55–59*	0.735	−0.001	0.005	[0.00 %]	0.313	−0.048	0.014	[0.27 %]
* 60–64*	0.079	0.069	0.048	[−1.33 %]	0.619	−0.040	0.047	[0.75 %]
* 65–69*	0.001**	0.117	−0.019	[0.90 %]	0.551	0.045	−0.021	[0.39 %]
* 70–74*	0.002*	0.091	−0.061	[2.25 %]	0.678	0.018	−0.064	[0.46 %]
* 75–79*	0.001**	0.092	−9.233	[8.53 %]	0.492	0.035	−0.234	[3.30 %]
* 80+*	0.001**	0.091	−9.337	[12.19 %]	0.617	0.037	−0.338	[4.97 %]
Education	-	-	-	8.99 %	-	-	-	2.97 %
* (R = Higher education)*	-	-	-	-	-	-	-	-
* Lower education*	0.187	0.127	−0.257	[13.12 %]	0.329	0.030	−0.260	[3.14 %]
* Middle*	0.275	0.030	0.340	[−4.13 %]	0.464	0.001	0.337	[−0.17 %]
Income	-	-	-	45.06 %	-	-	-	42.40 %
* (R = Not difficult on present income)*	-	-	-	-	-	-	-	-
* Difficult on present income*	0.000**	0.360	−0.314	[45.06 %]	0.001**	0.338	−0.313	[42.40 %]
Level of social activity	-	-	-	11.11 %	-	-	-	10.72 %
* (R = Average or higher than others in the same age)*	-	-	-	-	-	-	-	-
* Lower than others in the same age*	0.002*	0.148	−0.188	[11.11 %]	0.002*	0.144	−0.186	[10.72 %]
Self-assessed social position *(ordinal. 9 points. low to high)*	0.027*	−0.549	0.050	11.00 %	0.037*	−0.543	0.050	10.98 %
Labor market status	---	---	---	---	-	-	-	22.37 %
* (R = On labor market)*	---	---	---	---	-	-	-	-
* Permanently disabled*	---	---	---	---	0.003*	0.015	−0.183	[1.07 %]
* Retired*	---	---	---	---	0.001**	0.455	−0.117	[21.31 %]

*p = < 0.05, **p = < 0.001

We ran two decomposition models, attempting to explain observed health inequality by its association with the selected covariates. In both models, we included factors known to associate with health and health inequality; the only difference is that Model 1 did not contain information about labor-market status. Model 2 fills this gap, and was better fitted than Model 1, given smaller residue and higher R^2^. There was only one difference between the two models: in the absence of labor-market status age explained more than 21 % of socioeconomic health inequality. However, when data regarding work, retirement, and disability were included (i.e., Model 2), age became less relevant, explaining 9 % of health inequality (and non-significant). Retired people were particularly likely to report poor health, and even more likely than those who did not participate in the labor market due to disability and long-lasting illnesses (the value of elasticity was 0.455 versus 0.015). Both groups were not only more likely to report poor health than those active in the labor market, but also concentrated in the lower socioeconomic strata, contributing to a considerable portion of the observed health inequalities (22 %). In the Model 2 a simple, binary distinction concerning financial difficulties explained 42 % of overall health inequality. Educational differences alone did not substantially associate with health inequalities, but social activity did. People who were less active assessed their health worse than those more socially active (elasticity = 0.144), and they generally belonged to lower socioeconomic groups (CI = −0.186). This variable contributed 11 % of overall health inequality. The last covariate in the model was subjective social position. The overlap between subjective and objective measures of socioeconomic position was modest (CI = 0.05, Pearson’s correlation R = 0.265, *p* = <0.001) and slightly lower than reported in the literature [51]. High socioeconomic position predicted health outcomes, as the elasticity of −0.543 indicates, and its contribution to inequalities was 11 %.

Altogether, the contribution of demographic factors to health inequalities was only 9.5 %, compared to 89.5 % contribution of the social factors.

## Discussion

Contrary to a common sense perception that health decline is caused by biological ageing, this study shows that social consequences of older age, such as income, exit from the labor market, level of social activity and self-assessment of social position mediate large proportion of health deterioration of ageing people. Controlling for these social characteristics removes the association between poor self-assessed health and age in the population aged 45 and more years old. Similarly, results of the inequality decomposition also show that poor health is concentrated in the lower socioeconomic layers of society and that the most influential, individual contributors to the observed health inequalities are social in nature, rather than demographic.

We will now discuss some mechanisms that contribute to observed socioeconomic inequalities in health which were identified in the course of this study.

### Age and retirement

We estimated that the contribution of age to observed health inequalities at 10 % and showed that the relationship between age and health are not monotonous. Other studies using the same technique report a whole range of results pertaining to the association between health, health inequalities and age.. Sözmen et al. [34] take note of the statistically significant decline in self-rated health in mid-fifties in the Turkish population but estimates the overall contribution of age to health inequalities at only 4.9 %. Other studies which use very different health measures and look at an adult population report a 7.6 % contribution in New Zealand and 19.2 % contribution in Australia [54], 11.5 % in Canada (age and sex combined) [38], 19 % contribution in England [37] and 23 % contribution in Teheran, Iran [35]. It is very difficult to compare these figures given the differences in the health indicator, the socioeconomic indicator, and the list of covariates. These studies, however, do not include a detailed information about the type of labor market involvement which turned out to be so relevant in our sample. We, show that being retired associates with a drop in self-assessment of health, even when controlling for age and income associated with change in economic status due to leaving employment. Simultaneously, retired, elderly people are concentrated in lower layers of the society. The combination of these two characteristics results in one-fifth of socioeconomic health inequality in a population of Polish people aged 45 or over being attributed to retirement alone. Gundgaard and Lauridsen also performed a decomposition of health inequalities including, among many other covariates, age and labor market situation. They found out that the retirement, with its 18.5 % contribution to health inequalities in the Danish population aged 18–60, is the strongest single predictor of General Health, as measured by the SF-36 scale [55]. Its contribution to health inequalities turned out to be even higher –up to 64.5 %—when health in the population aged 18–60 was measured using the EQ-5D scale [56]. Two explanations of these findings about the labour market situation mediating much of the health decline observed in older cohorts are salient. First, retirement can result from health problems, especially among early retirees [27, 29]. In Poland, approximately 1 in 5 people who exit the labor market is motivated by poor health [57], and the pattern is probably more prevalent among workers with lower skills and salaries, given health is generally worse in lower socioeconomic layers. Such workers might be more likely to seek retirement as a strategy for avoiding unemployment, an explanation that favors social selection. The second explanation is grounded in the social-causation perspective, assuming that retirement has causal consequences to health. Socially advantaged people might, on average, enjoy more control over the course of their careers [58] and therefore more likely to postpone retirement or adopt proactive strategies of labor-market exit. This is important, given the state’s policy in Poland in the 1990s and early 2000s, to push older workers out of the labor market for the benefit of younger generations [42]. Those less educated and modestly rewarded might have experience such forced disengagement, which is on its own detrimental to workers’ health [21, 59, 60], more frequently than their socioeconomically advantaged counterparts. On the top of that, these disadvantaged workers might have comparably fewer resources which could aid retirement adaptation, such as financial means and networks promoting social engagement.

### Education

In this study we noted an educational gradient in health which was almost fully mediated by other covariates used in the analysis, such as labor market participation income, social engagement and self-esteem. Similarly, educational differences seemed to play a role for health inequalities only in the absence of the information about the labor market participation. In such a model, the contribution of education to health inequalities was 9 %, which is comparable or even higher that in other similar studies [37, 39, 61]. However, once the labor market information was added, the contribution of education to health inequalities shrank to 3 % (not statistically significant). This might likely stem from the fact that education to a large extent determines both occupational chances and choices which are so relevant for health. Two other studies which also performed a decomposition of health inequalities including age, education and labor market situation as covariated (albeit with different indicators of health) noted a contribution of education to overall health inequalities in the Danish population aged 16–80 years old at 4 [55] and 7 % [56].

### Subjective SES as a proxy for social inferiority

Although both objective and subjective socioeconomic positions are strong predictors of health status, they do not correlate highly. Objective and subjective SES might denote different components of a socioeconomic position, and therefore feed disparate mechanisms, translating socioeconomic standing into health outcomes. Epidemiological studies rarely acknowledge that socioeconomic status is a multifaceted concept, encompassing not only material circumstances (measured by income) and general cognitive aptitude (measured by education), but also social prestige and social distress. In our model, although income related strongly to health and health inequalities, it did not explain the range of complexity of how social hierarchy influences health. We propose that subjective SES, particularly with control for income and education, is treated as a proxy for perceptions of social inferiority, particularly significant in highly unequal societies [62], such as Poland. Subjective perceptions of one’s position in a social hierarchy might influence the psychosocial pathway especially strongly [50, 63]. This proposition explains evidence from longitudinal studies, in which low subjective social status predicted functional declines in older adults, even after adjustments for objective components of socioeconomic positions and health statuses at a baseline of 4 years prior [64]. Of course, the social-selection explanation also applies here, suggesting part of the association stems from a mechanism of reversed causation (i.e., a negative influence of health issues on both socioeconomic position and self-esteem). We consider this inability to distinguish cause and effect a limitation of the study, which we mitigate by addressing the literature. All associations reported in this study can be explained rationally in terms of social causation and social selection, the two primary paradigms of social epidemiology. Another limitation derives from the properties of the analytical technique. Decomposition of a CI is an arithmetic tool that does not evaluate the direction of the relationship, nor does it test whether all variables were included. We tested the model repeatedly to ensure reliability and validity, and found that the relative contribution of income and age remained the same regardless of how the variables were included in the model (e.g., binary, dummy, and numeric). We also found that variables such as marital status and religious involvement, included commonly in social epidemiology research, did not relate to health inequalities. Finally, we did not include insurance status as a covariate potentially contributing to health inequalities because it does not differentiate the chances for receiving health care in Poland, where the public social insurance scheme financed from a mandatory quasi-tax provides free of charge primary and specialist care to all citizens in education, labor force or retirement [65]. In fact, the information about the insurance status is not even routinely collected in surveys, including the ESS used for the purpose of this study. The factor which most strongly differentiates the actual health care utilization is whether or not an individual has the financial capacities to purchase outpatient medicines and pay out-of-pocket health services from a private health-care sector which is dynamically growing in response to inefficiencies of the public one [66]. We included income information in our analyses and noted that the financial capabilities are indeed very relevant for health.

A final limitation originates from incomplete data which had to be deleted listwise before decomposition.

## Conclusions

This study analyzes factors associated with self-reported health and health inequalities in along the socioeconomic hierarchy among people aged 45 and over, based on data from Poland. We show a socioeconomic gradient in self-assessed health of the middle-age and older Polish population. Yet, age contributes to no more than 10 % of this observed inequality, and is by far not the primary source of health inequality observed at the societal level. Labor market situation, and particularly retirement, is the second strongest single contributor to health inequality (after income) in this age group. Other important contributors are subjective socioeconomic position (as a measure of social inferiority) and the level of social activity. Educational level does not have a direct contribution to health and health inequalities as long as the other aforementioned are also taken into account. However, no cross-sectional study can affirm the ordering and causal mechanisms that link covariates with health inequalities; longitudinal studies are necessary to resolve that.

The findings imply critical importance of social factors for health and health inequalities, with major consequences for social policy. Public policy targeting older groups needs to structurally address health disparities and provide systemic opportunities for maintaining good health. Policy-makers need to acknowledge that capacities of older workers might differ depending on socioeconomic status and type of occupation which has particular applications in most Western economies in which postponing the retirement age is high on political agenda. Such pension reforms, although necessary due to population aging, should be accompanied with actions that minimize negative consequences, mitigating health declines (i.e., healthy aging) and promoting age management in companies that maintain the work ability of older workers and adjust work environments to their requirements.

We acknowledge that there are multiple mechanisms responsible for associations between socioeconomic position and health. Three common transmission mechanisms can generally be distinguished: the effect of socioeconomic deprivation on health, the effect of social inferiority on health, and the effect of health on socioeconomic position. We argue that all played a role in this study, though the last with much less force than the previous two.

## Abbreviations

CI: Concentration Index
ESS: European Social Survey
SAH: Self-Assessed Health
SES: Socioeconomic status.
